# Dentine hypersensitivity and associated factors: a Nigerian cross-sectional study

**DOI:** 10.11604/pamj.2019.33.272.18056

**Published:** 2019-07-30

**Authors:** Kofoworola Olaide Savage, Olabisi Hajarat Oderinu, Adeleke Oke Oginni, Omolara Gbonjugbola Uti, Ilemobade Cyril Adegbulugbe, Oluwole Oyekunle Dosumu

**Affiliations:** 1 Department of Preventive Dentistry, Faculty of Dental Sciences, College of Medicine, University of Lagos, Lagos, Nigeria; 2 Department of Restorative Dentistry, Faculty of Dental Sciences, College of Medicine, University of Lagos, Lagos, Nigeria; 3 Department of Restorative Dentistry, Faculty of Dentistry, Obafemi Awolowo University, Ile-Ife, Ife-ife, Nigeria; 4 Department of Restorative Dentistry, Faculty of Dentistry, College of Medicine, University of Ibadan, Ibadan, Nigeria

**Keywords:** Dentine hypersensitivity, prevalence, associated factors, erosion, tooth brushing

## Abstract

**Introduction:**

Prevalence of dentine hypersensitivity (DH) may be on the increase as a result of changing lifestyles. This study aimed to assess the prevalence of DH and relative importance of associated factors in 18-35 year old Nigerians and compare to findings from a similar European study.

**Methods:**

Following ethical approval, 1349 subjects from the six geopolitical zones in Nigeria participated in this cross sectional study. DH was clinically evaluated by cold air tooth stimulation, patient pain rating (yes/no) and investigator rated pain using the Schiff ordinal scale (0-3). Erosive tooth wear using the BEWE index was assessed. A questionnaire regarding the nature of the DH, erosive dietary intakes, tooth brushing habits and other factors was completed by patients. Bivariate analysis was conducted.

**Results:**

32.8% of patients reported pain on tooth stimulation and 32.9% scored ≥1 on Schiff scale for at least one tooth. Questionnaire reported sensitivity was 41.2%. There were statistically significant associations between Schiff score and clinically elicited DH (p < 0.001); and BEWE erosive tooth wear score and clinically elicited DH (p < 0.001). There were significant associations between DH and some oral hygiene practices such as brushing frequency, brush movement and brushing after breakfast. Fresh fruit and fruit/vegetable juice intake also showed significant association.

**Conclusion:**

The most important risk factors of DH for this population in Nigeria appear to be the frequency and characteristics of tooth brushing. This should be considered in its prevention and management.

## Introduction

Dentine Hypersensitivity (DH) is characterized by short sharp pain arising from exposed dentine in response to thermal, evaporative, tactile, osmotic or chemical stimuli that cannot be ascribed to any other dental defect or disease. It is an exaggerated response to a sensory stimulus that usually cause no response in a normal healthy tooth [1]. Other possible causes of pain that should be eliminated before a diagnosis of DH is made include fractured or chipped teeth, carious lesions, palatogingival grooves, leaky restorations and cracked cusps [2]. Dentinal pain is mediated by a hydrodynamic mechanism [3]. A pain provoking stimulus applied to dentine increases the flow of dentinal tubular fluids, this mechanically activates the nerves situated at the inner ends of the tubules. The pain thus initiated is often associated with mild to severe discomfort which often affects patients' eating and drinking habits [1], hence affecting their quality of life. It has been reported that cold stimulus is more effective in activating intradental nerves than do heat and probing [4, 5]. This is supported by the observation that close to 75% of patients with DH complain of pain from cold stimuli [6]. The prevalence of DH varies from 1.34% to 98% [7, 8]. Although DH may affect patients of any age group, it mostly occurs in patients who are between 30 and 40 years old [2], overall review of literature shows equal gender. Different distribution patterns have been reported [9], canines and premolars are most often affected [6, 10] however, it may affect any tooth. DH condition starts with exposure of dentine by the loss of enamel and or gingival recession (with loss of cementum), this has been termed ´lesion localisation´. The exposure of root dentine secondary to gingival recession has been reported to be associated with overzealous tooth brushing [11], about 70% of people suffering from DH brush more than twice daily [12]. Not all exposed dentine is sensitive, there must be the opening of the dentinal tubule system to permit activation of the hydrodynamic mechanism by appropriate stimuli, termed ´lesion initiation´. This occurs when the smear layer and or tubular plugs are removed, which opens the outer ends of the dentinal tubules [13]. Abrasion and more importantly, dietary acid erosion may be implicated [14]. DH is more frequently encountered in patients with periodontal diseases [9, 15]. Hypersensitivity has been reported to occur in about half of patients after periodontal procedures such as deep scaling, root planing and gingival surgery [16]. DH may also occur in non-carious cervical lesions especially when exposed to erosive foods and drinks. Although several risk factors leading to the exposure of dentine, tubular opening and subsequent pain have been identified, their relative importance has been controversial. DH is likely to increase in prevalence for a number of reasons; increase in life expectancy, retention of teeth throughout life, changing life styles notably diet, change from traditional African diet to western diet in urban city dwellers, and increased intake of fizzy drinks as seen in developing African countries. It was therefore the objectives of this study to determine by questionnaire combined with clinical examination the prevalence of DH and its associated factors in 18-35 years old Nigerians and to compare the findings to a similar study carried out in 18-35 years old Europeans [17].

## Methods

Nigeria is divided into six geopolitical zones each comprising of states that share similar culture, ethnic groups and common history. The zones are North Central, North East, North West, South East, South South and South West. Not all the states in each zone were identified to have public dental hospitals or clinics in either urban or rural locations. For this reason, in order to effectively perform the clinical examination protocol for this study, seven states, each representing a geopolitical zone and Federal Capital Territory (Abuja) where both rural and urban dental facilities are available were included. Adults aged 18-35 years from seven states representing the six geopolitical zones in Nigeria and the Federal Capital Territory (Abuja) were recruited. These participants were recruited from patients attending designated dental centres in each of the seven states and the Federal Capital Territory during the study period. Two centres located in rural/small-middle sized town and metropolitan city in each of the seven states were used. The sample size exceeded the calculated minimum sample for DH prevalence based on previously reported DH prevalence of 1.34% among a Nigerian population [7) and further included the number of participant recruited within specified study duration (6 months) and this improved the power of the study. Ethical approval for the study was obtained from the Medical Ethics Committee of the Lagos University Teaching Hospital (LUTH). Oral and written consent to participate was obtained from all patients after a comprehensive explanation of the study in local languages where applicable. The data reported in this study was part of a larger national study patterned after the European Study in Non Carious Cervical Lesions (Escarcel). Escarel [18] is a Pan European study designed to estimate the levels of sensitivity, periodontal disease and tooth wear in young adults. After screening, consenting patients who met inclusion criteria were recruited. Patients were required to be healthy, between 18 and 35 years of age, and able to follow all study procedures and restrictions. Exclusion criteria included; patients with 5 teeth or less, currently having orthodontic appliances, cervical restorations, taking analgesics, or undergone oral local anaesthesia in the last 24 hour, people requiring antibiotics for dental treatment, on anticoagulants or who suffered bleeding disorders, or were employee of the study centre. Examiners calibration was organized by 3 members of the Escarcel group. Intra- and inter examiner reliability was evaluated. The Kappa agreement among all the examiners at the end of the training phase was 85.5%. A self-administered questionnaire based on the one used for the European study was completed by each participant. The questionnaire included data on risk factors associated with non-carious cervical lesions (use of tobacco, medication, erosive dietary factors) general lifestyle, dietary and oral health behaviour, perception of dentine hypersensitivity including intensity, duration and origin. Following completion of the questionnaire, a clinical examination for dentine hypersensitivity, erosive tooth wear and loss of periodontal attachment was performed. All eligible teeth excluding the second and third molars were assessed for presence or absence of DH, erosive tooth wear and periodontal loss of attachment.

The exposed dentine surface of each eligible tooth was subjected to cold air stimulation by a one second application of air from the air spray of the dental unit or a triple air dental syringe from a distance of approximately 10 mm with adjacent teeth shielded. The patient´s response to the cold air stimulation was recorded by the examiner using the Schiff ordinal scale [19]: (0 = subject does not respond to stimulus, 1 = subject respond to stimulus but does not request discontinuation of stimulus, 2 = subject respond to stimulus and request discontinuation or moves away from stimulus, 3 = subject respond to stimulus, considers stimulus to be painful, and request discontinuation of stimulus). The patient was then asked whether the stimulus provoked DH or not. This procedure was undertaken for each eligible tooth in turn. Non-carious cervical lesions were evaluated using the Basic Erosive Wear Examination (BEWE) on the facial/buccal, lingual/palatal surfaces using an ordinal scale (0 = no erosive wear, 1 = early tooth loss, 2 = surface loss <50%, 3 = wear with tissue loss >50% of the surface) [20]. The location of the lesion (coronal surface, root surface or crown-root junction) was recorded. Bivariate statistical analysis was carried out at the patient level. Elicited sensitivity was related to several categorical variables. Odds ratios were reported in relation to the appropriate categorical variables, with 95% confidence intervals. The relationships between the measures of sensitivity i.e. DH on any tooth on cold air stimulation, Schiff score and questionnaire declared hypersensitivity; and of elicited sensitivity to tooth wear and recession were also analysed. Non-carious cervical lesions were evaluated using the Basic Erosive Wear Examination (BEWE) on the facial/buccal, lingual/palatal surfaces using an ordinal scale (0 = no erosive wear, 1 = early tooth loss, 2 = surface loss <50%, 3 = wear with tissue loss >50% of the surface) [19]. The location of the lesion (coronal surface, root surface or crown-root junction) was recorded. Bivariate statistical analysis was carried out at the patient level. Elicited sensitivity was related to several categorical variables. Odds ratios were reported in relation to the appropriate categorical variables, with 95% confidence intervals. The relationships between the measures of sensitivity i.e. DH on any tooth on cold air stimulation, Schiff score and questionnaire declared hypersensitivity; and of elicited sensitivity to tooth wear and recession were also analysed.

## Results

In all, 1349 adults were recruited. The mean number of teeth evaluated for DH in each subject was 23.7 (range 19-24). The mean number of teeth with DH was 6.36 (range 0-18). Data analysed was based on number (n) that responded to the variable of interest in the questionnaire. Table 1 shows the proportions of patients having DH according to the three measures of sensitivity. 443 patients (32.8%) reported DH in at least one of the teeth evaluated in response to cold air stimulation. A maximum Schiff score of 3 was recorded for 64 patients (4.7%), while in 220 patients (16.3%) and 444 patients (32.9%) a Schiff score of 2 or 3 and 1 or higher were recorded respectively. Out of the 1349 patients who completed the DH question in the questionnaire, 556 (41.2%) reported DH. These respondents were then asked how important the pain was to them. 550 responded to this question, out of which 151 (27.5%) said the pain was ''very important'' (95% C.I. 23.6% to 31.5%) Table 2 shows that there was a statistically significant association between self-reported hypersensitivity and clinically elicited sensitivity (p < 0.001); Schiff score and clinically elicited DH (p < 0.001). This table also shows the association of elicited DH with erosive tooth wear. There were significant associations between elicited DH and erosive tooth wear (p < 0.001. There was a closer relationship between maximum BEWE score and elicited sensitivity. Table 3 shows the relationship of elicited DH to a range of subject's associated demographic factors. While Table 4 shows only subjects' associated oral hygiene and dietary factors that had significant association. Statistically significant associations were found between elicited sensitivity and some socio-demographic characteristics like age, area of residence (rural or urban), and level of education (p < 0.001). Some oral hygiene factors such as brush frequency, brush movement, brushing after breakfast were statistically associated with elicited sensitivity. Also, elicited sensitivity was statistically associated with fresh fruit intake and fruit /vegetable juice intake (p < 0.001). Other life-style factors such as smoking, use of certain medications, snoring and chewing gum did not show statistical significance (Annex 1).

**Table 1:** Prevalence of hypersensitivity by 3 criteria

			95% confidence intervals	
	Number	Percentage	Lower	Upper
Total patients	1349			
**DH any tooth on cold air stimulation (Clinical elicited DH)**				
Yes	443	32.8	30.2%	35.4%
No	906	67.2		
**Schiff highest score**				
0	905	67.1	64.5%	69.5%
1	224	16.6	14.7%	18.6%
2	156	11.6	10.0%	13.3%
3	64	4.7	3.4%	5.9%
2-3	220	16.3	14.3%	18.3%
1-3	444	32.9	30.5%	35.4%
**Self-reported hypersensitivity**				
Yes	556	41.2	38.6%	43.9%
No	709	52.6		
Not sure	84	6.2		

**Table 2:** Relationship between three measures of sensitivity, and of elicited sensitivity to tooth wear

		Elicited Sensitivity			Odds	95% Confidence Limits		Chi	df	P-value
	n	Yes		Percent (%)	Ratio	Lower	Upper	Square		
**Total patients**	1349	443		32.8%						
**Schiff highest score**										
0	905	93		10.3%	0.031	0.022	0.042	640.058	3	<0.001*
1	224	177		79.0%	12.161	8.572	17.254			
2	156	130		83.3%	14.058	9.050	21.836			
3	64	43		67.2%	4.530	2.653	7.735			
**Self-reported hypersensitivity**										
Yes	556	217		39.0%	1.606	1.276	2.021	16.483	2	<0.001*
No	709	203		28.6%	0.669	0.532	0.840			
Unknown/not sure	84	23		27.4%	0.759	0.463	1.243			
**Tooth wear – BEWE score**										
0	537	47		8.8%	0.10	0.07	0.14	276.50	3	<0.001*
1	279	95		34.1%	1.07	0.81	1.42			
2	397	223		56.2%	4.26	3.32	5.47			
3	136	78		57.4%	3.12	2.18	4.48			
									

* = Statistically significant

**Table 3:** Bivariate analyses for relationship of elicited sensitivity to demographic factors

		Elicited Sensitivity		Odds Ratio	95% Confidence Limits		Chi Square	df	P-value
	n	Yes	(%)	(OR)	Lower	Upper	X^2^		
Total Patients	1349	443	32.8%						
**Age (yrs)**	1303								
18 - 25	466	133	28.5%	0.738	0.578	0.942	6.81	2	0.033*
26 - 35	837	291	34.8%	1.262	0.996	1.600			
**Gender**	1329								
Male	592	184	31.1%	0.867	0.689	1.091	1.44	1	0.240
Female	737	252	34.2%	1.145	0.911	1.440			
**Centre**									
Osun	200	32	16.0%	0.342	0.230	0.509	87.25	7	<0.001*
Oyo	200	29	14.5%	0.301	0.199	0.454			
Edo	100	34	34.0%	1.057	0.687	1.625			
Enugu	100	38	38.0%	1.276	0.838	1.943			
Kano	200	81	40.5%	1.478	1.086	2.012			
Lagos	250	97	38.8%	1.378	1.037	1.831			
FCT	200	95	47.5%	2.080	1.534	2.821			
Borno	99	37	37.4%	1.239	0.811	1.893			
**Area of Residence** 1147									
Rural	395	106	26.8%	0.672	0.518	0.870	7.95	2	0.019*
Small/Mid-size towns	100	27	27.0%	0.741	0.469	1.170			
Metropolitan zone	652	226	34.7%	1.173	0.935	1.473			
**Education**	828								
To age 15^+^	265	106	40.0%	1.478	1.120	1.950	15.68	3	<0.001*
To age 16 – 19	106	38	35.8%	1.156	0.764	1.750			
To age 20^+^	185	73	39.5%	1.399	1.016	1.925			
Still studying	272	69	25.4%	0.639	0.473	0.863			
**Occupation**	1238								
Self employed	201	60	29.9%	0.850	0.613	1.178	10.28	6	0.113
Managers	28	7	25.0%	0.677	0.285	1.604			
Other white collars	335	117	34.9%	1.133	0.873	1.470			
Manual workers	61	18	29.5%	0.850	0.484	1.492			
House person	101	41	40.6%	1.438	0.950	2.177			
Unemployed	97	34	35.1%	1.112	0.721	1.716			
Student	415	114	27.5%	0.696	0.540	0.898			

* = Statistically significant. OR=1; factor does not have effect on elicited sensitivity, OR>1; factor associated with high odds elicited sensitivity, OR<1; factor associated with lower odds of elicited sensitivity

**Table 4:** Bivariate analyses for relationship of elicited sensitivity to oral hygiene and dietary antecedent factors

	n	Elicited Sensitivity		Odds Ratio	95% Confidence Limits		Chi Square	df	P-value
		Yes	(%)	(OR)	Lower	Upper	X2		
Total Patients	1349	443	32.8%						
Brushing Frequency	1265								
Once per day	1009	308	30.5%	0.667	0.517	0.861	10.16	2	0.006*
Twice per day	247	101	40.9%	1.537	1.157	2.042			
Thrice per day	9	2	22.2%	0.582	0.120	2.815			
Brush Movement	1329								
Various motion	403	140	34.7%	1.130	0.883	1.445	10.41	4	0.034*
Horizontal	334	115	34.4%	1.100	0.847	1.428			
Vertical	517	151	29.2%	0.763	0.602	0.967			
Circular	53	26	49.1%	2.030	1.170	3.522			
Don’t know/Not sure	22	7	31.8%	0.954	0.386	2.356			
**Brush after breakfast**									
Often	437	109	24.9%	0.575	0.446	0.742	37.42	4	<0.001*
Occasionally	215	104	48.4%	2.197	1.634	2.955			
Rarely	240	79	32.9%	1.004	0.746	1.352			
Never	303	106	35.0%	1.132	0.865	1.482			
Don’t know	154	45	29.2%	0.827	0.572	1.194			
**Fresh fruits**									
Often	390	143	36.7%	1.272	0.993	1.628	13.34	4	0.010*
Occasionally	754	237	31.4%	0.866	0.689	1.088			
Rarely	154	44	28.6%	0.798	0.551	1.155			
Never	24	4	17.4%	0.425	0.144	1.258			
Don’t know	27	15	55.6%	2.611	1.212	5.627			
**Fruit/Vegetable juice**									
Often	340	139	40.9%	1.604	1.243	2.069	19.79	4	<0.001*
Occasionally	711	213	30.0%	0.759	0.604	0.953			
Rarely	234	70	29.9%	0.849	0.625	1.153			
Never	44	10	23.3%	0.611	0.298	1.251			
Don’t know	20	11	55.0%	2.538	1.044	6.170			

* = Statistically significant. OR=1; factor does not have effect on elicited sensitivity, OR>1; factor associated with high odds elicited sensitivity, OR<1; factor associated with lower odds of elicited sensitivity

**Annex 1:** Bivariate analyses for relationship of elicited sensitivity to oral hygiene, dietary and personal antecedent factors

	n	Elicited Sensitivity		Odds Ratio	95% Confidence Limits		Chi Square	df	P-value
		Yes	(%)	(OR)	Lower	Upper	X^2^		
Total Patients	1349	443	32.8%						
Brushing Frequency	1265								
Once per day	1009	308	30.5%	0.667	0.517	0.861	10.16	2	0.006*
Twice per day	247	101	40.9%	1.537	1.157	2.042			
Thrice per day	9	2	22.2%	0.582	0.120	2.815			
Toothbrush used	1265								
None	21	4	19.0%	0.476	0.159	1.425	6.40	4	0.171
Manual toothbrush	1193	395	33.1%	1.114	0.776	1.598			
Electric toothbrush	26	7	26.9%	0.750	0.313	1.796			
Chewing stick	18	2	11.1%	0.252	0.058	1.102			
Others	7	3	42.9%	1.538	0.343	6.899			
Brush Movement	1329								
Various motion	403	140	34.7%	1.130	0.883	1.445	10.41	4	0.034*
Horizontal	334	115	34.4%	1.100	0.847	1.428			
Vertical	517	151	29.2%	0.763	0.602	0.967			
Circular	53	26	49.1%	2.030	1.170	3.522			
Don’t know/Not sure	22	7	31.8%	0.954	0.386	2.356			
Brush after breakfast									
Often	437	109	24.9%	0.575	0.446	0.742	37.42	4	<0.001*
Occasionally	215	104	48.4%	2.197	1.634	2.955			
Rarely	240	79	32.9%	1.004	0.746	1.352			
Never	303	106	35.0%	1.132	0.865	1.482			
Don’t know	154	45	29.2%	0.827	0.572	1.194			
Brush before breakfast									
Often	1004	342	34.1%	1.248	0.956	1.629	7.08	4	0.132
Occasionally	195	57	29.2%	0.822	0.590	1.145			
Rarely	72	21	29.2%	0.834	0.495	1.405			
Never	70	18	25.7%	0.696	0.402	1.204			
Don’t know	8	5	62.5%	3.436	0.817	14.443			
Brush after lunch									
Often	40	12	30.0%	0.873	0.440	1.734	5.89	4	0.208
Occasionally	72	22	30.6%	0.895	0.535	1.497			
Rarely	533	162	30.4%	0.831	0.658	1.051			
Never	687	238	34.6%	1.182	0.941	1.484			
Don’t know	17	9	52.9%	2.328	0.892	6.075			
Brush after dinner									
Often	385	139	36.1%	1.227	0.957	1.573	6.61	4	0.158
Occasionally	306	109	35.6%	1.175	0.899	1.535			
Rarely	299	92	30.8%	0.885	0.671	1.168			
Never	330	96	29.1%	0.795	0.606	1.041			
Don’t know	29	7	24.1%	0.645	0.273	1.522			
Snoring									
Often	88	29	33.0%	1.006	0.635	1.593	4.37	4	0.359
Occasionally	155	45	29.0%	0.818	0.567	1.181			
Rarely	306	113	36.9%	1.265	0.969	1.651			
Never	587	193	32.9%	1.003	0.798	1.262			
Don’t know	213	63	29.6%	0.836	0.607	1.150			
Sleeping medication/antidepressant									
Often	23	10	43.5%	1.586	0.690	3.647	3.77	4	0.439
Occasionally	64	21	32.8%	0.999	0.585	1.705			
Rarely	235	83	35.3%	1.144	0.851	1.537			
Never	991	321	32.4%	0.927	0.718	1.197			
Don’t know	36	8	22.2%	0.577	0.261	1.276			
Smoking									
Often	50	20	40.0%	1.381	0.775	2.460	3.00	4	0.558
Occasionally	90	28	31.1%	0.918	0.579	1.457			
Rarely	162	60	37.0%	1.235	0.878	1.737			
Never	1030	329	31.9%	0.671	0.647	0.970			
Don’t know	17	6	35.3%	1.117	0.410	3.041			
Chew gum									
Often	191	63	33.0%	1.008	0.728	1.396	2.28	4	0.685
Occasionally	580	200	34.5%	1.139	0.906	1.432			
Rarely	333	103	30.9%	0.890	0.682	1.162			
Never	216	70	32.4%	0.977	0.716	1.333			
Don’t know	29	7	24.1%	0.645	0.273	1.522			
Acidic foods									
Often	345	128	37.1%	1.304	1.010	1.684	5.47	4	0.243
Occasionally	591	190	32.1%	0.946	0.752	1.190			
Rarely	280	88	31.4%	0.922	0.695	1.223			
Never	109	31	28.4%	0.799	0.518	1.231			
Don’t know	23	5	21.7%	0.563	0.208	1.527			
Fresh fruits									
Often	390	143	36.7%	1.272	0.993	1.628	13.34	4	0.010*
Occasionally	754	237	31.4%	0.866	0.689	1.088			
Rarely	154	44	28.6%	0.798	0.551	1.155			
Never	24	4	17.4%	0.425	0.144	1.258			
Don’t know	27	15	55.6%	2.611	1.212	5.627			
Fruit/Vegetable juice									
Often	340	139	40.9%	1.604	1.243	2.069	19.79	4	<0.001*
Occasionally	711	213	30.0%	0.759	0.604	0.953			
Rarely	234	70	29.9%	0.849	0.625	1.153			
Never	44	10	23.3%	0.611	0.298	1.251			
Don’t know	20	11	55.0%	2.538	1.044	6.170			
Isotonic/energy drinks									
Often	81	34	42.0%	1.519	0.962	2.399	3.64	4	0.457
Occasionally	342	113	33.1%	1.018	0.784	1.322			
Rarely	384	126	32.8%	0.998	0.776	1.284			
Never	502	157	31.3%	0.893	0.705	1.131			
Don’t know	40	13	32.5%	0.984	0.503	1.927			
Soft drinks									
Often	361	113	31.3%	0.909	0.701	1.177	8.22	4	0.084
Occasionally	679	237	34.9%	1.208	0.962	1.517			
Rarely	222	67	30.2%	0.863	0.632	1.180			
Never	67	16	23.9%	0.628	0.354	1.115			
Don’t know	20	10	50.0%	2.302	0.929	5.706			
Dairy products									
Often	167	59	35.3%	1.135	0.808	1.595	0.97	4	0.914
Occasionally	570	189	33.2%	1.025	0.815	1.290			
Rarely	446	144	32.3%	0.963	0.756	1.227			
Never	126	38	30.2%	0.872	0.585	1.299			
Don’t know	40	13	32.5%	1.023	0.521	2.011			

* Statistically significant. OR=1; Factor does not have effect on elicited sensitivity, OR>1; Factor associated with high odds elicited sensitivity, OR<1; Factor associated with lower odds of elicited sensitivity.

## Discussion

This clinical and questionnaire based cross sectional study among young Nigerian adults to determine the prevalence of DH and its associated factors, presents data among public hospital attending participants just as the European study by West *et al* [17]. These participants can be said to represent young Nigerian adults of varied ethnic, cultural, economic status, occupation and balanced rural and urban dwellers. The inclusion and exclusion criteria further eliminated bias towards the disease condition studied. The present study suggests that about one in every three young adult Nigerian (32.8%) may have dentine hypersensitivity as determined by responses to cold air stimulation in a clinical setting. This is relatively low in comparison with a similar European study by West *et al* [17] that reported a prevalence of 41.9%. But comparison to findings from other previous clinical studies in Nigeria; 1.34% [7], 16.3% [21], in Europe; 2.8% [22] and in Australia 9.1% [23], the reported prevalence of the present study (32.8%) was very high. Particularly, the higher prevalence of DH recorded in this study when compared to previous clinical studies [7,21] among Nigerian population, suggest that dentine hypersensitivity may be on the increase in our environment. The clinical prevalence of DH (32.8%, 32.9%) versus self-reported DH (41.2%) in this present study further support reports that prevalence data obtained from questionnaires based studies were often a little higher than that obtained by clinical examination [24-26]. It has been suggested that the majority of patients demonstrated some coping mechanisms for dealing with pain as shown by the findings of the European study where peoples' perception of their pain is less than that of clinical reporting [17]. This is contrary to the findings of the current study where peoples' perception of their pain is more than that of clinical reporting. However, a sizeable percentage (27.5%) in the present study felt that the pain intensity was ''very important'' to their lifestyle, this should be put in proper perspective when considering the treatment need for this condition and its impact on the quality of life. There was no differences in the prevalence of DH according to gender in the present study and the European study [17]. Similar studies [24-26] have reported the same findings, while others [27,28] have reported a female preponderance. This study finding corroborate the observation from the European study that the clinical elicited method of assessing DH correlate with the Schiff score for pain of DH. Also, there were significant associations between elicited sensitivity after stimulation and erosive wear which reinforced the similar findings reported in the European study [17]. A range of potential associated factors to DH were assessed in this study. The results showed a significant association of DH with tooth brushing frequency, and brushing after breakfast. More than 60% of participants brushed their teeth 2 or 3 times daily. These associations may also be due to the erroneous believe that the harder the tooth brush and force of brushing, the cleaner the teeth becomes. A combination of these factors will definitely lead to loss of dental hard tissue with dentine exposure. Brushing after breakfast will further enhance the hard dental tissue loss due to dietary acid challenge. In contrast to our findings, the frequency and characteristics of tooth brushing were not significantly associated with DH in the European study [17]. Rather, erosive dietary factors played significantly in the DH experienced by the young European studied [17].

## Conclusion

The prevalence of DH in young Nigerian adults (18-35years) is low compared to their European counterparts. Dentine hypersensitivity may be on the increase and most important risk factors for dentine hypersensitivity among young Nigeria adult population appear to be the frequency and characteristics of tooth brushing. This should be considered in its prevention and management.

### What is known about this topic

- Dentine hypersensitivity is a distinct clinical phenomenon whereby dentine is exposed and reactive;
- Dentine hypersensitivity have been associated to oral hygiene and acidic dietary risk factors.

### What this study adds

- Important risk factors for dentine hypersensitivity is different among populations.

## Competing interests

The authors declare no competing interests.
